# Investigating the Effects of a Manuka Honey, Royal Jelly, and Bee Venom-Derived Face Serum on Skin Health and Signs of Aging

**DOI:** 10.7759/cureus.81244

**Published:** 2025-03-26

**Authors:** Ellen O'Gorman, Swathi Varanasi, Scott Bukoski, Susanne Mitschke, Scott Conger

**Affiliations:** 1 Clinical Research, Citruslabs, Las Vegas, USA

**Keywords:** dark spots, dry skin, fine lines, skincare, wrinkles

## Abstract

Objective: The purpose of this study was to investigate the efficacy of a commercially available manuka honey-based face serum that includes royal jelly and bee venom on various ingredients frequently associated with skin health benefits over an eight-week period.

Materials and methods: Forty female participants aged 40-55 with self-reported skin health concerns were recruited. Participants used the serum twice daily and completed questionnaires during Weeks 2, 4, and 8. Photos of the face were analyzed for dermatological skin grading and Optic Elite facial analysis at Week 8.

Results: There were significant improvements in fine lines, wrinkles, dark spots, hyperpigmentation, dryness, and overall skin health beginning at Week 2, with sustained enhancements observed until Week 8. Dermatologist skin grading results were mixed, with 20 (60.6%) of participants demonstrating improvements in skin brightness, but lower percentages of participants showed improvement in overall skin health, fine lines/wrinkles, roughness, pigmentation, and redness/erythema. Optic Elite analysis showed improvements in several skin health scores, providing further evidence for the serum’s effects on skin health. Participants self-reported high satisfaction with the effectiveness of the serum.

Conclusions: These findings suggest that the face serum may be an effective skincare product for improving skin health and mitigating signs of aging.

## Introduction

Facial skin health significantly influences perceptions of age and attractiveness. The appearance of forehead lines and upper facial lines is often aesthetically perceived as “unattractive” and increasing concerns about looking older than the actual age [1]. Effective skin care interventions can play an important role in one’s overall quality of life. Many people gravitate toward interventions that use naturally derived products. One such combination is skin care treatments that utilize manuka honey with royal jelly and bee venom.

Honey has a long history of use as a skincare product. During the first century, honey was included in various recipes for face packs [2]. In medieval times, honey was often used as a skin moisturizer [3]. Honey is naturally acidic and contains high levels of hydrogen peroxide, leading to antibacterial properties on the skin [4]. Furthermore, its high levels of carbohydrates and fruit acids may yield regenerative effects by increasing tissue oxygenation and elimination of harmful metabolites [4]. Honey also has hygroscopic properties leading to detoxification of dermal tissue. This increases skin tension, improves skin elasticity, and smooths wrinkles in the skin [4]. Fruit acids in honey also result in an exfoliating effect for dead skin cells [4].

Manuka honey is distinct from other types of honey due to its unique chemical composition and potential health benefits. It has a stronger antibacterial effect than other types of honey because of larger amounts of methylglyoxal (MGO) [5] and phenolic compounds [6,7]. More specifically, it exhibits potent antimicrobial activity against pathogens such as *Staphylococcus aureus* and *Helicobacter pylori*, suggesting potential efficacy in combating acne-causing bacteria on the skin [8,9]. Traditionally used in wound healing, manuka honey promotes tissue regeneration and aids in the recovery of minor cuts, burns, and abrasions [10,11].

Moreover, manuka honey serves as a natural humectant, drawing moisture into the skin, aiding in hydration, and has anti-inflammatory properties to soothe irritated skin and alleviate redness [12,13]. In addition to manuka honey, royal jelly and bee venom are both recognized for their beneficial properties in skincare, offering advantages such as antibacterial, anti-inflammatory, moisturizing, and anti-aging properties [14]. Bee venom is often used in products that are used to treat photoaging [15] and acne [16].

Royal jelly is also an effective ingredient in cosmetics for skin care problems. In nature, royal jelly is used to feed the queen bee for her lifetime and bee larvae during the first three days of life [17]. As a skincare ingredient, royal jelly improves regenerative processes in the skin. This is particularly useful in acne-prone skin where lesions and small wounds often occur [18]. Royal jelly is highly moisturizing and increases the hydration of the stratum corneum by retaining water in it, increasing the elasticity of the skin [19]. Royal jelly has been shown to have many beneficial properties including anti-inflammatory, antioxidative, antimicrobial, and anti-tumoral [17,20,21]. Previous research has also found royal jelly to have anti-bacterial and immune regulatory effects [17,22]. Royal jelly has also exhibited anti-aging properties by inhibiting cellular degradation activities and enhancing collagen synthesis [23]. Therefore, the royal jelly ingredient in the face serum used in the present study may have had an additive effect on the skin health of the participants.

This study aimed to examine the efficacy of a face serum that contains manuka honey, royal jelly, and bee venom on facial skin health. This face serum will be tested with daily use over the course of eight-weeks in a population of volunteers with concerns about their skin health.

## Materials and methods

Participants

A total of 40 participants living in Southern California were recruited for this study. Participant demographics are presented in Table 1. Participants were informed of the potential risks and benefits of the study and signed an independent review board (IRB) approved informed consent form prior to enrolling. The study was approved by an IRB, adhered to all Good Clinical Practice guidelines in accordance with the Helsinki Declaration, and was registered with clinicaltrials.gov (ID: NCT6148766).

**Table 1:** Characteristics of Participants Values are reported as the sample size and percentage of the total sample or as mean±standard deviation.

Characteristics	Values
Ethnicity, n (%)	
White individuals	27 (67.5)
Asians	4 (10)
Black individuals	2 (5)
Hispanic individuals	5 (12.5)
Native Americans	1 (2.5)
Not reported	1 (2.5)
Fitzpatrick Scale Skin Type, n (%)	
Type 1	6 (15)
Type 2	23 (57.5)
Type 3	9 (22.5)
Type 4	1 (2.5)
Type 5	1 (2.5)
Age (yrs)	48±5.0
Height (m)	1.65±0.06
Weight (kg)	74.8±19.8
BMI (kg·m-2)	27.7±7.7

Participants who were included in this study met the following criteria: female-at-birth, between the ages of 40 and 55, generally healthy with no known chronic disease, concerned with overall skin health and appearance, including fine lines/wrinkles and the appearance of dark spots, use of the same cleanser, toner, and moisturizer for at least one month prior to starting the study and willing to continue the same skin regime for the duration of the study, not currently using oral or topical retinoids, and did not introduce any new medications or supplements that target skin health in the three months prior to beginning the study. Participants were excluded if they met any of the following criteria: allergy to bees or bee products, pregnant or currently breastfeeding, individuals with cystic acne or otherwise self-reported very acne-prone skin, use of a prescription medication relevant to the skin, any cosmetic procedures during the study, including Botox, laser, or chemical peel treatments, sensitivity or allergy to any ingredients found in the products, or had any severe chronic conditions, including oncological conditions, psychiatric disorders, or diabetes. All participants satisfied the inclusion and exclusion criteria.

Investigational product

Each participant in this study used the Manuka Health Eternal Renewal Regenerating Face Serum with Royal Jelly and Bee Venom (Manuka Health, Auckland, New Zealand). This product contains MGO equal to or greater than 800 milligrams per kilogram of manuka honey. MGO is a naturally occurring compound found in manuka honey, and it is one of the key components responsible for its unique antibacterial properties. The product also contains royal jelly, bee venom, and a proprietary blend of other skincare ingredients including hyaluronic acid and botanical extracts such as iris root, myrtle, bay laurel leaf, beetroot, and rose fruit. The Eternal Renewal Regenerating Face Serum is designed for topical application as part of a daily skincare routine.

Study design

Following the consent process, participants completed the Baseline self-administered questionnaire (see Appendix A), and a selection of participants attended a Baseline Optic Elite facial analysis visit. The Optic Elite facial analysis was completed using the CatchMD CAPTURE system (CatchMD, Dallas, Texas, USA). The CatchMD CAPTURE system employs a combination of advanced imaging modalities to conduct detailed skin analyses. Utilizing RGB (red, green, blue) imaging, the system captures high-resolution surface-level photographs, while polarized light (PL) imaging minimizes surface reflections to enhance visualization of subsurface vascular structures and pigmentation irregularities. Ultraviolet (UV) imaging highlights subclinical damage caused by UV exposure, revealing conditions not visible under standard lighting. The system processes these images through integrated analytical software, which quantifies skin parameters such as wrinkle depth, pore size, texture irregularities, pigmentation, and UV damage. Questionnaires were completed again after Week 2, Week 4, and Week 8. The Optic Elite facial analysis was conducted after Week 8 on the subset of participants who completed this assessment at Baseline. All participants provided an endline photo of their face for expert skin grading.

All participants also provided a Baseline photo of their face for expert skin grading by a dermatologist. The photos were taken of the full face/head from an angle directly in front of the participant's face with hair tied back so that it was not covering the face. Natural light was used without any shadows on the face and all photos were taken at the same time of the day. Participants were asked to take the photo without makeup, without using the camera flash, and with a neutral facial expression. The dermatologist analyzed photos of participants’ faces at Baseline and Week 8. These photos were graded on improvement from the Baseline on several parameters, including overall skin quality, fine lines/wrinkles, roughness, pigmentation, redness/erythema, and brightness. Dermatologist evaluations were conducted using a binary "yes/no" grading system to assess improvements across six parameters, providing a straightforward measure of visible changes.

Participants were asked to apply the investigational product each morning and before bedtime at night. After thoroughly cleansing and toning the face, the participants applied two to three drops of the serum onto clean, dry fingers and massaged over the entire face until fully absorbed. The product was then followed with the participant’s preferred moisturizer.

Statistical analysis

Unless indicated otherwise, means and standard deviations (SD) were calculated for all data. Data from the questionnaires were collected using a textual 5-point Likert scale for each question. The textual Likert data was transformed into numerical values from 1 (worst outcome) to 5 (best outcome). Data were checked for normality using the Pearson test. A repeated measures analysis of variance (RM ANOVA) was used to compare participant outcomes at each check-in to their Baseline response. Data were analyzed using a mixed-effects analysis test based on the normality of the data, with Dunnett corrections for multiple comparisons. For the CatchMD Optic Elite analysis, a percentile measurement was calculated for each skin health parameter, which indicated the value below which a given percentage of observations in a group of observations falls. The 100th percentile was a perfect score. For the data collected from the Optic Elite, individual paired t-tests were conducted for each analyzed parameter. Statistical analyses were performed using GraphPad Prism 9.0 (GraphPad Software, Boston, MA, USA), and the significance level was set at an alpha level of 0.05. For product-specific questions evaluated only on Week 2, Week 4, and Week 8, results were presented as % of subjects reporting each answer.

## Results

Clinical evaluation of the face serum on skin health parameters

The effects of the face serum on 17 skin health parameters were evaluated at Baseline, Week 2, Week 4, and Week 8. At Week 2, 12 of 17 parameters were significantly improved (p<0.05) from the Baseline, including crow’s feet, lip lines, dark spots, hyperpigmentation, pore size, complexion, dryness, perception of skin plumpness, suppleness, hydration, and softness, skin redness, glow, and overall health (Table 2). Questionnaire responses at Week 4 demonstrated significant improvement in 16 out of 17 parameters tested when compared with Baseline (p<0.05). The additional improvements seen in Week 4 included fine lines, smile lines, forehead wrinkles, and skin texture (Table 2). By Week 8, all 17 skin parameters were significantly improved compared to the Baseline (p<0.01).

**Table 2:** Clinical Evaluation of the Face Serum on Skin Health Parameters For each question, RM ANOVAs were used to compare participant outcomes across all time points. The overall treatment effect F-values and p-values for each question are reported. When appropriate, post-hoc analysis with Dunnett corrections for multiple comparisons was used to compare Baseline values to each time point. The post-hoc p-values are reported for each comparison. An increase in score indicates an improvement. RM ANOVA: repeated measures analysis of variance

Question	Overall Treatment Effect (F-value)	Overall Treatment Effect (p-value)	Baseline (n=40) (mean±SD)	Week 2 (n=36) (mean±SD)	Base vs. Week 2 Post-hoc (p-value)	Week 4 (n=36) (mean±SD)	Base vs. Week 4 Post-hoc (p-value)	Week 8 (n=37) (mean±SD)	Base vs. Week 8 Post-hoc (p-value)
How would you rate your fine lines?	10.10	<0.01	3.10±0.55	3.17±0.56	0.81	3.44±0.61	<0.01	3.54±0.61	<0.01
How would you rate your wrinkles?	7.769	<0.01	3.25±0.59	3.36±0.59	0.59	3.42±0.65	0.15	3.70±0.70	<0.01
How would you rate your crow’s feet (fine lines & wrinkles around the eye area)?	10.32	<0.01	3.03±0.73	3.33±0.72	0.04	3.47±0.74	<0.01	3.62±0.68	<0.01
How would you rate your smile/marionette lines (around the mouth)?	7.722	<0.01	3.00±0.78	3.14±0.87	0.63	3.31±0.71	0.02	3.60±0.69	<0.01
How would you rate your lines/wrinkles around your lips (specifically around your upper lip)?	10.99	<0.01	3.48±0.78	3.86±0.68	<0.01	3.83±0.70	<0.01	3.95±0.71	<0.01
How would you rate the lines/wrinkles on your forehead?	5.883	<0.01	3.03±0.83	3.33±0.76	0.09	3.42±0.73	<0.01	3.54±0.80	<0.01
How would you rate your dark spots?	9.034	<0.01	3.30±0.69	3.69±0.82	<0.01	3.72±0.70	<0.01	3.87±0.82	<0.01
How would you rate your hyperpigmentation (dark patches of skin)?	8.861	<0.01	3.53±0.82	3.94±0.83	<0.01	4.03±0.74	<0.01	4.05±0.85	<0.01
How would you rate your pore size?	11.66	<0.01	2.95±0.88	3.36±0.87	0.02	3.58±0.73	<0.01	3.57±0.77	<0.01
How would you rate your skin's texture?	15.54	<0.01	2.95±0.68	3.22±0.59	0.06	3.47±0.56	<0.01	3.60±0.64	<0.01
How satisfied are you with your skin complexion (the natural color, texture, and appearance of your skin)?	27.65	<0.01	2.43±0.64	2.86±0.87	<0.01	3.42±0.94	<0.01	3.60±0.86	<0.01
How would you rate the dryness of your skin?	12.10	<0.01	3.13±0.85	3.53±0.97	<0.01	3.58±0.84	<0.01	3.92±0.72	<0.01
Would you describe your skin as plump and hydrated?	19.43	<0.01	2.23±0.66	2.61±0.93	0.04	2.94±0.79	<0.01	3.16±0.69	<0.01
Would you describe your skin as soft and supple?	20.08	<0.01	2.28±0.75	2.75±0.91	<0.01	3.06±0.79	<0.01	3.22±0.71	<0.01
How would you rate the redness of your skin?	10.72	<0.01	3.43±0.98	3.81±0.92	0.01	3.97±0.81	<0.01	4.03±0.93	<0.01
Would you describe your skin as glowing?	24.81	<0.01	1.80±0.85	2.50±1.00	<0.01	2.67±0.89	<0.01	3.08±0.89	<0.01
How would you rate the overall health of your skin?	13.00	<0.01	3.08±0.53	3.36±0.54	0.03	3.64±0.54	<0.01	3.62±0.64	<0.01
How would you rate the elasticity of your skin?	7.928	<0.01	2.88±0.65	3.03±0.65	0.55	3.33±0.72	<0.01	3.35±0.68	<0.01

Participants’ perception of the face serum: insights from user feedback

**Figure 1:**
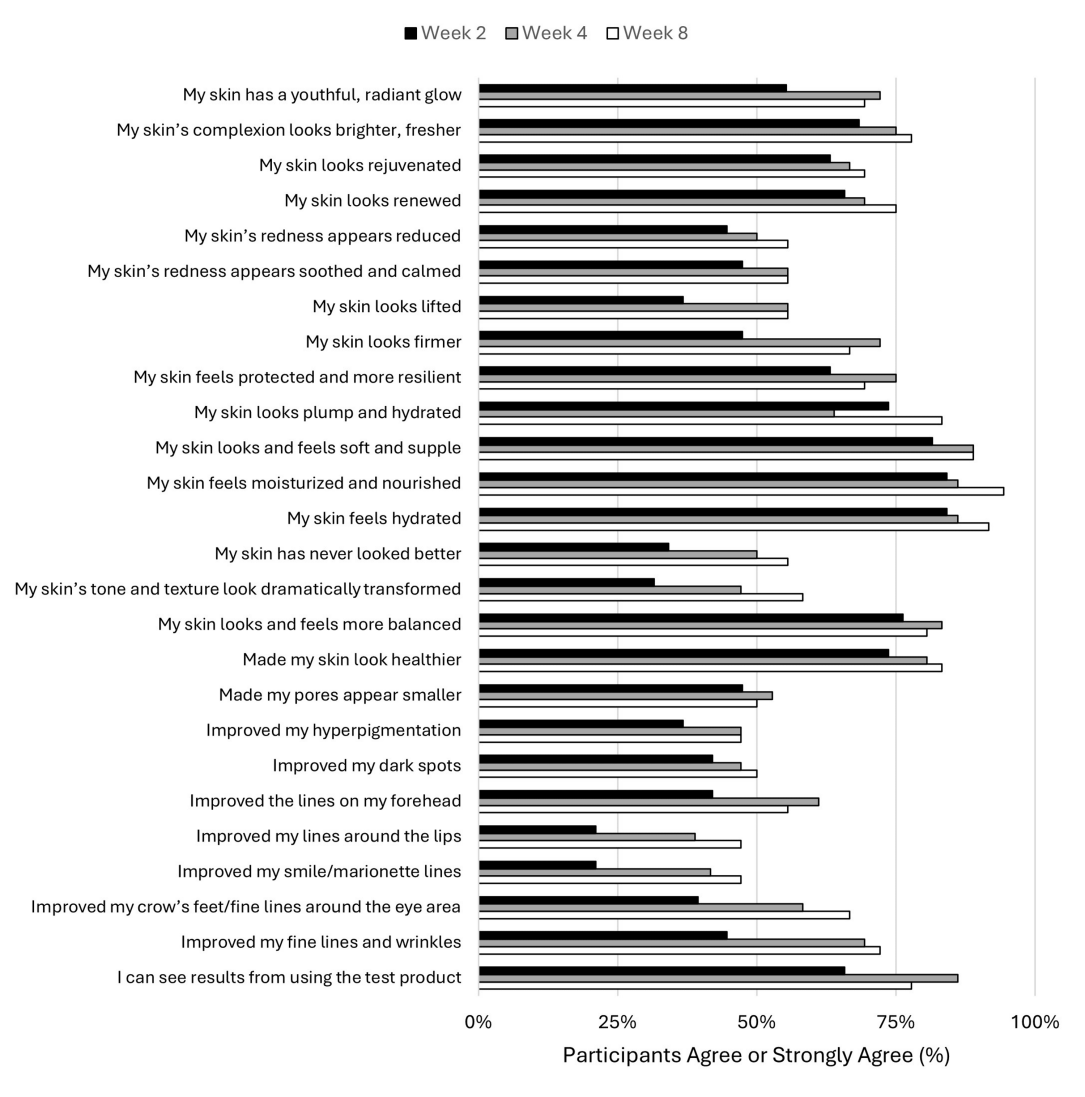
Participants’ Perception of the Face Serum: Insights From User Feedback

Participants were asked to respond to questions at the end of Week 2, Week 4, and Week 8, which provided insight into the participants’ perceptions of the face serum. Participants responded to face serum evaluation questions with a “strongly agree” to “strongly disagree” scale. The “strongly agree” and “agree” responses were combined into a single “combined agree” result to evaluate the overall participant perception. Consistent with the data analyses presented in Table 2, ≥70% of participants agreed that the test product made their skin look healthier, more balanced, hydrated and plump, moisturized and nourished, and soft and supple by the end of Week 8. These were maintained from Week 2 (Figure 1). By Week 8, 9 of 26 parameters had the agreement of ≥75% of participants. These included healthier skin (n=31, 83.3%), skin balance (n=30, 80.6%), overall hydration (n=34, 91.7%), skin hydration and plumpness (n=31, 83.3%), moisturization and nourishment (n=35, 94.4%), softness and suppleness (n=33, 88.9%), skin renewal (n=28, 75%), skin complexion (n=29, 77.8%), visible results (n=29, 77.8%) (Figure 1).

Dermatologist face analysis

**Figure 2:**
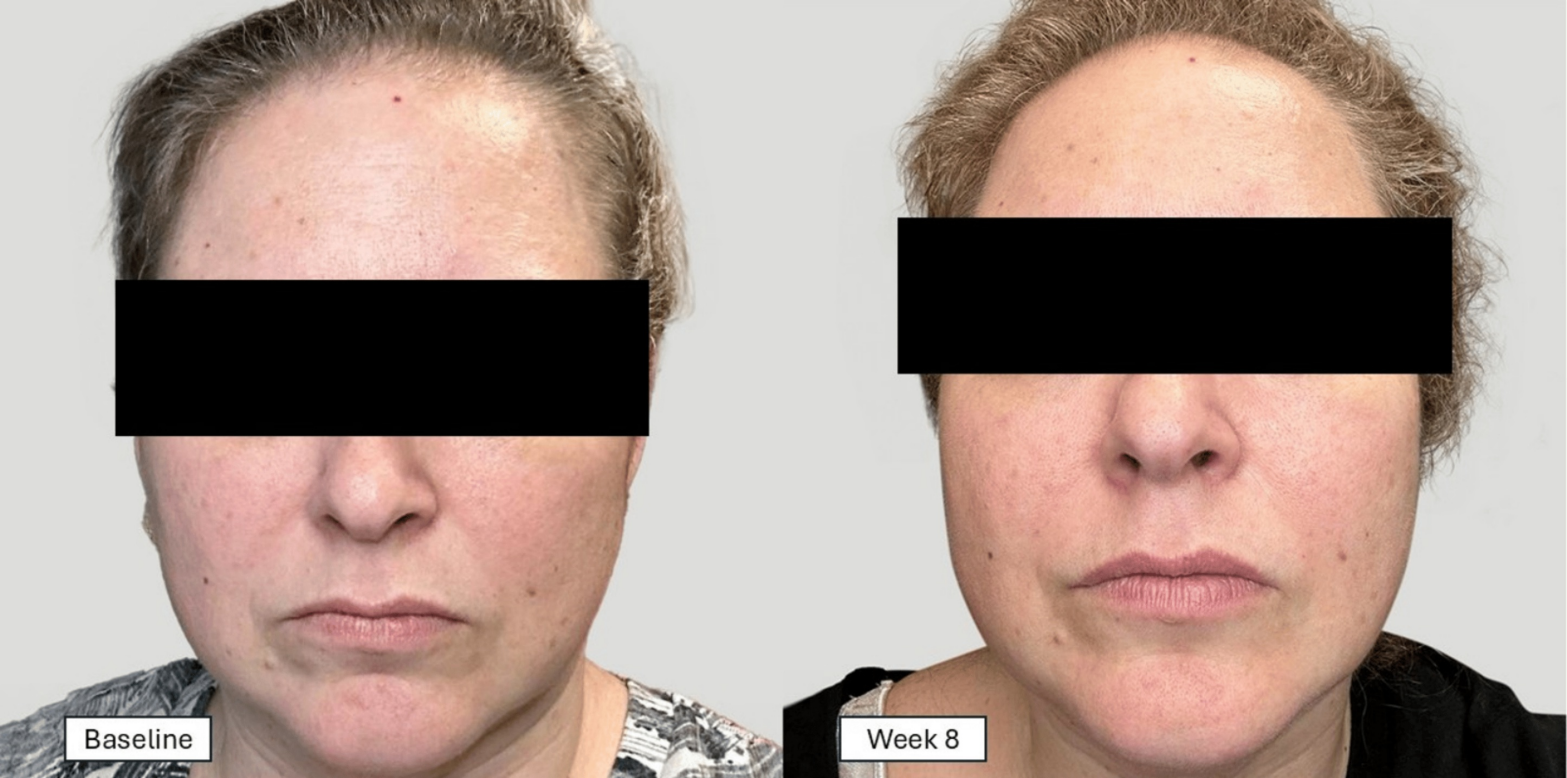
Example of a Facial Skin Photograph Used for Skin Grading by the Dermatologist The patient has provided written informed consent, and any accompanying images have been published.

Figure 2 presents a representative example of the skin photos that were used by the dermatologist for the determination of improvements. When comparing skin photos from Baseline to Week 8, the dermatologist grading demonstrated notable improvement in skin brightness in 20 (60.6%) of the participants. The other five parameters showed improvement ranging from 5 (15.2%) to 11 (33.3%) of the participants (Table 3).

**Table 3:** Dermatologist Assessment of Facial Skin Photographs - Comparison of Baseline and Week 8 Using a binary yes/no grading system, the dermatologist was asked to assess if each parameter improved when compared to the Baseline photo. The extent of improvement was not evaluated.

Parameter	Proportion of Participants That Improved From Baseline, n (%)
Overall skin quality	9 (27.3)
Fine lines/wrinkles	7 (21.2)
Roughness	11 (33.3)
Pigmentation	5 (15.2)
Redness/erythema	9 (27.3)
Brightness	20 (60.6)

Optic Elite analysis

The Optic Elite imaging system captures a series of three distinct photographic images, each employing a different lighting modality to enhance diagnostic precision. The RGB image integrates red, green, and blue lighting to produce a full-color image that accurately represents surface skin characteristics. The UV image highlights fluorescence patterns, aiding in the identification of features that contrast with surrounding healthy skin. Finally, the PL image minimizes surface shine, providing a clear, unobstructed view of dermal structures, vascular conditions, inflammation, and pigmentation. With this, the Optic Elite analysis evaluated 10 skin health parameters: RGB Pore, RGB Spot, RGB Wrinkles, PL Texture, UV Porphyrin, UV Pigmentation, UV Moisture, Sensitive Areas, Brown Area, and UV Damage. A subsample of 10% of the participants (the first four participants who were enrolled in the study) completed the Optic Elite analysis.

**Figure 3:**
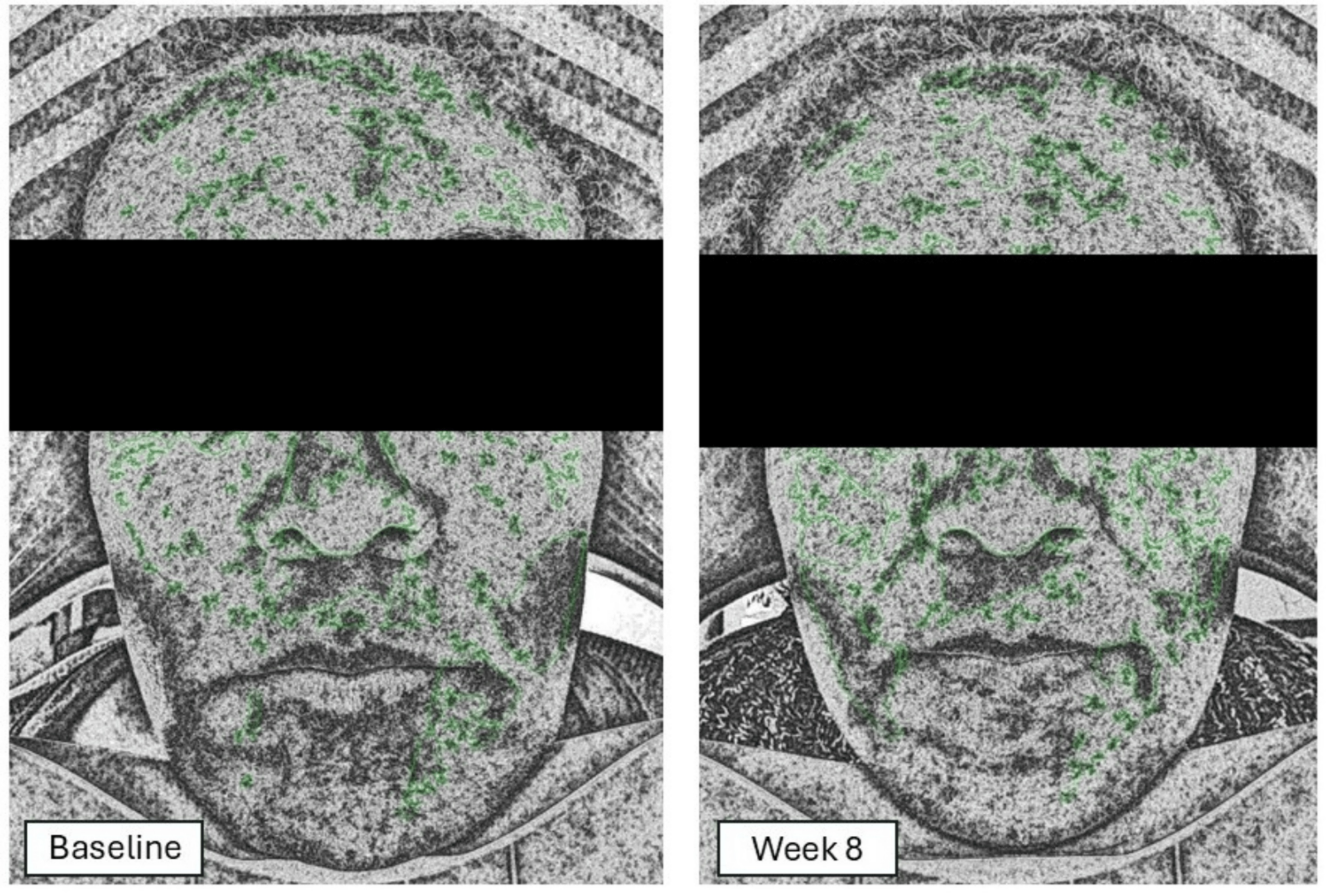
Example of a UV Exposure Image Generated for a Participant During Optic Elite Analysis UV: ultraviolet The patient has provided written informed consent, and any accompanying images have been published.

Figure 3 presents an example of the images that were generated during the Optic Elite analysis. None of the parameters demonstrated statistically significant improvements. However, 7 of 10 parameters demonstrated non-significant improvement from the Baseline in Optic Elite scores, including RGB Pore, RGB Spot, RGB Wrinkles, PL Texture, Sensitive Area, Brown Area, and UV Damage (Table 4). The most improved parameter was RGB Wrinkle, which increased by 7.5 percentage points (44.25-51.75%). However, the mean pigmentation score decreased by 4.5 percentage points (46.25-41.75%), which correlates with the dermatologist’s scoring.

**Table 4:** CatchMD Optic Elite Analysis of Skin Health Parameters The data are reported as the mean percentile of a group (100 = 100th percentile). The p-values were determined using individual paired t-tests of the mean percentile values for each analyzed parameter. RGB: red, green, blue; PL: polarized light; UV: ultraviolet

Parameter	Baseline (n=4)	Endline (n=4)	P-value	T-value
RGB Pore	30±6.32	33.5±7.85	0.28	1.315
RGB Spot	33.25±9.64	39.75±13.10	0.25	1.421
RGB Wrinkle	44.25±11.67	51.75±16.13	0.27	1.353
PL Texture	32±8.45	36.5±11.09	0.31	1.214
UV Porphyrin	36.5±6.95	34.75±8.77	0.76	0.329
UV Pigmentation	46.25±3.77	41.75±9.57	0.49	0.792
UV Moisture	26.5±6.56	28.75±11.15	0.63	0.527
Sensitive Area	40.5±10.34	46.75±14.03	0.28	1.321
Brown Area	33±7.70	36±14.09	0.60	0.579
UV Damage	33.5±5.45	34±14.31	0.95	0.072

## Discussion

This study evaluated the impact of a face serum that contained royal jelly and bee venom on various skin health parameters over an eight-week period. The findings demonstrated significant and rapid improvements in multiple skin health parameters as measured from subjective questionnaire data. Specifically, by Week 2 patients perceived improvements in 12 out of 17 parameters assessed including crow’s feet, lip lines, dark spots, hyperpigmentation, pore size, complexion, dryness, perception of skin plumpness, suppleness, hydration, and softness, skin redness, glow, and overall skin health. This suggests that the participants’ perception of the face serum exerts its effect quickly and improves a variety of skin health parameters. Furthermore, the sustained improvements observed across all skin health parameters by Week 8 highlight the serum’s potential to deliver lasting benefits. Notably, self-perceived enhancements of skin glow were 71% compared with the Baseline (Table 2), suggesting the serum’s efficacy in promoting overall skin radiance and vitality over the study duration.

Previous research has used bee venom as a cosmetic ingredient. Because of its antibacterial and anti-inflammatory effects, it can be used as an ingredient in anti-acne products [4]. Bee venom has also demonstrated the ability to restore cell damage and collagen production after ultraviolet B radiation (UVB) exposure. This protection from the effects of UVB exposure may prevent or reduce skin wrinkling [24,25]. Han et al. [26] evaluated the effects of a bee venom serum on facial wrinkles in a sample of 22 women. In this study, the participants were also asked subjective questions about the impact of the bee venom serum on variables such as skin moisture, skin elasticity, skin appearance, and wrinkles. After 12 weeks, 50% or more participants agreed or strongly agreed with only three of the eight questions. These questions included “makes my skin look healthier and more youthful” (54.6%), “improves the appearance of wrinkles” (54.6%), and “improves the softness of my skin” (50%). In the present study, after eight weeks of use participants agreed or strongly agreed that “the test product improved my fine lines and wrinkles” (72.2%), “the test product makes my skin look healthier” (83.3%), “my skin looks and feels soft and supple after using the product” (88.9%), “my skin feels hydrated after applying the product” (91.7%), and “my skin feels moisturized and nourished after using the product” (94.4%). Han et al. [26] also indicated that the bee venom serum decreased total wrinkle area, total wrinkle count, and wrinkle depth. While there were no significant improvements in the objective measures of the skin as assessed by the dermatologist or using the CatchMD Optic Elite analysis, the face serum used in the present study seemed to be more well-received by the participants than in the study by Han et al. [26].

There were inherent limitations to the study design that should be considered in examining the expert skin grading results. To perform the skin grading, the experts were only provided pictures taken at certain time points. Despite standardizing the pictures, unavoidable variability between the images (e.g. lighting, overall image quality) could have obscured improvements in dark spots during comparisons. Moreover, day-to-day skin fluctuations could also mean that a participant’s dark spots appeared worse on the day of the picture than any of the days before or after. Measuring this outcome could, therefore, favor the participants’ self-reported results. By observing their skin daily, the participants may be better able to identify incremental improvements in their dark spots, and they would also be aware of natural fluctuations in their skin. However, as they do not possess the knowledge of the experts, the participants are less capable of providing an accurate assessment.

The dermatologist analysis of photos at Baseline and Week 8 provided some insight into the effect of the product on skin health. At Week 8, only a small percentage of participants were observed to improve several parameters. This may indicate that the significant effects observed in the patient-reported outcomes may be due to specific participants observing considerable improvements. Alternatively, it is possible that the participants’ perception of change was more notable than the expert skin grading objective assessment. The notable percentage of participants experiencing skin brightness does, however, indicate a visible positive effect of the serum over the eight weeks.

There were several strengths in this study. The study utilized a decentralized trial design. The participants were asked to use the product with limited restrictions in a real-world setting outside of the laboratory. The sample size of this study was also larger than similar studies in this area. In addition, the CatchMD Optic Elite device provided much greater detail of the skin than the photos that were used to assess changes in the skin parameters by the dermatologist.

There were also several limitations that were inherent in the design of this study. The study was designed as a single-group study, lacking a placebo or control group for comparison. This makes it difficult to attribute the changes that are reported in this study solely due to the test product. As is the case with any single-group study that utilizes self-report measures, there is the potential for bias as participants’ responses may have been influenced by the expectations of improvement. The objective assessments by the dermatologist were completed by assessment of photographs taken by the participants. Despite standardized methods, these photographs may not fully capture subtle improvements due to potential variability in lighting, image quality, or daily skin fluctuations. In addition, the dermatologist was not blinded and the binary grading system could have been expanded to allow for more descriptive improvements over time. A more robust study design would utilize in-person assessments with quantitative evaluation of skin parameters. While the sample size was larger than similar studies in this area, a total sample size of 40 participants could be considered relatively small. The demographics of the study population were limited with more than 50% of the participants self-reporting as white and type 2 on the Fitzpatrick scale skin type. The CatchMD Optic Elite analysis was only completed on a small subset of the participants. Thus, the interpretation of the results from the CatchMD Optic Elite is limited due to insufficient statistical power. The discrepancy between the self-reported questionnaire results and the CatchMD Optic Elite analysis may be due to the differences in the number of participants who completed each measurement. The eight-week duration of the study could also be considered a limitation. In a systematic review of studies on the treatment of photoaging, the minimum duration of the included studies was 12 weeks [27]. A study duration of eight weeks may be insufficient for this type of study. Future studies should aim for targeted methods to recruit a more diverse study population and a larger sample of participants who completed the CatchMD Optic Elite analysis.

## Conclusions

This study demonstrated the potential of significant and rapid improvements in skin health parameters observed after using a facial serum containing manuka honey, royal jelly, and bee venom. The serum could offer a viable, consumer-friendly solution for addressing common skin concerns such as hydration, plumpness, and radiance, particularly for individuals seeking rapid results. This study lays the groundwork for future research to further elucidate the mechanisms related to manuka honey, royal jelly, and bee venom on skin health. Overall, this study suggests that this facial serum holds promise as an effective skincare intervention.

## Appendices

Appendix A

**Table 5:** Questionnaire Credit: Ellen O'Gorman

Question	Response Group
How would you rate your fine lines?	Not noticeable, Barely noticeable, Moderate Distracting, Severe
How would you rate your wrinkles?	Not noticeable, Barely noticeable, Moderate Distracting, Severe
How would you rate your crow’s feet (fine lines & wrinkles around the eye area)?	Not noticeable, Barely noticeable, Moderate Distracting, Severe
How would you rate your smile/marionette lines (around the mouth)?	Not noticeable, Barely noticeable, Moderate Distracting, Severe
How would you rate your lines/wrinkles around your lips (specifically around your upper lip)?	Not noticeable, Barely noticeable, Moderate Distracting, Severe
How would you rate the lines/wrinkles on your forehead?	Not noticeable, Barely noticeable, Moderate Distracting, Severe
How would you rate your dark spots?	Not noticeable, Barely noticeable, Moderate Distracting, Severe
How would you rate your hyperpigmentation (dark patches of skin)?	Not noticeable, Barely noticeable, Moderate Distracting, Severe
How would you rate your pore size?	Not noticeable, Barely noticeable, Moderate Distracting, Severe
How would you rate your skin's texture?	Very poor, Poor, Fair, Good, Excellent
How satisfied are you with your skin complexion (the natural color, texture, and appearance of your skin)?	Very satisfied, Satisfied, Neither satisfied nor dissatisfied, Dissatisfied, Very dissatisfied
How would you rate the dryness of your skin?	Not noticeable, Barely noticeable, Moderate Distracting, Severe
Would you describe your skin as plump and hydrated?	Extremely, Very much, Somewhat, A little bit, Not at all
Would you describe your skin as soft and supple?	Extremely, Very much, Somewhat, A little bit, Not at all
How would you rate the redness of your skin?	Not noticeable, Barely noticeable, Moderate Distracting, Severe
Would you describe your skin as glowing?	Extremely, Very much, Somewhat, A little bit, Not at all
How would you rate the overall health of your skin?	Very poor, Poor, Fair, Good, Excellent
How would you rate the elasticity of your skin?	Very poor, Poor, Fair, Good, Excellent
How much do you agree or disagree with this statement: I can see results from using the test product.	Strongly agree, Agree, Neither agree or disagree, Disagree, Strongly disagree
How much do you agree or disagree with this statement: The test product has improved my fine lines and wrinkles.	Strongly agree, Agree, Neither agree or disagree, Disagree, Strongly disagree
How much do you agree or disagree with this statement: The test product has improved my crow’s feet/fine lines around the eye area.	Strongly agree, Agree, Neither agree or disagree, Disagree, Strongly disagree
How much do you agree or disagree with this statement: The test product has improved my smile/marionette lines.	Strongly agree, Agree, Neither agree or disagree, Disagree, Strongly disagree
How much do you agree or disagree with this statement: The test product has improved my lines around the lips (especially the upper lip).	Strongly agree, Agree, Neither agree or disagree, Disagree, Strongly disagree
How much do you agree or disagree with this statement: The test product has improved the lines on my forehead.	Strongly agree, Agree, Neither agree or disagree, Disagree, Strongly disagree
How much do you agree or disagree with this statement: The test product has improved my dark spots.	Strongly agree, Agree, Neither agree or disagree, Disagree, Strongly disagree
How much do you agree or disagree with this statement: The test product has improved my hyperpigmentation.	Strongly agree, Agree, Neither agree or disagree, Disagree, Strongly disagree
How much do you agree or disagree with this statement: The test product has made my pores appear smaller.	Strongly agree, Agree, Neither agree or disagree, Disagree, Strongly disagree
How much do you agree or disagree with this statement: The test product makes my skin look healthier.	Strongly agree, Agree, Neither agree or disagree, Disagree, Strongly disagree
How much do you agree or disagree with this statement: My skin looks and feels more balanced after using the test product. (Neither dry nor oily)	Strongly agree, Agree, Neither agree or disagree, Disagree, Strongly disagree
How much do you agree or disagree with this statement: My skin’s tone and texture look dramatically transformed after using the test product.	Strongly agree, Agree, Neither agree or disagree, Disagree, Strongly disagree
How much do you agree or disagree with this statement: My skin has never looked better.	Strongly agree, Agree, Neither agree or disagree, Disagree, Strongly disagree
How much do you agree or disagree with this statement: My skin feels hydrated after applying the test product.	Strongly agree, Agree, Neither agree or disagree, Disagree, Strongly disagree
How much do you agree or disagree with this statement: My skin feels moisturized and nourished after using the test product.	Strongly agree, Agree, Neither agree or disagree, Disagree, Strongly disagree
How much do you agree or disagree with this statement: My skin looks and feels soft and supple after using the test product.	Strongly agree, Agree, Neither agree or disagree, Disagree, Strongly disagree
How much do you agree or disagree with this statement: My skin looks plump and hydrated after using the test product.	Strongly agree, Agree, Neither agree or disagree, Disagree, Strongly disagree
How much do you agree or disagree with this statement: My skin feels protected and more resilient after using the test product.	Strongly agree, Agree, Neither agree or disagree, Disagree, Strongly disagree
How much do you agree or disagree with this statement: My skin looks firmer after using the test product.	Strongly agree, Agree, Neither agree or disagree, Disagree, Strongly disagree
How much do you agree or disagree with this statement: My skin looks lifted after using the test product.	Strongly agree, Agree, Neither agree or disagree, Disagree, Strongly disagree
How much do you agree or disagree with this statement: My skin’s redness appears soothed and calmed after using the test product.	Strongly agree, Agree, Neither agree or disagree, Disagree, Strongly disagree
How much do you agree or disagree with this statement: My skin’s redness appears reduced after using the test product.	Strongly agree, Agree, Neither agree or disagree, Disagree, Strongly disagree
How much do you agree or disagree with this statement: My skin looks renewed after using the test product.	Strongly agree, Agree, Neither agree or disagree, Disagree, Strongly disagree
How much do you agree or disagree with this statement: My skin looks rejuvenated after using the test product.	Strongly agree, Agree, Neither agree or disagree, Disagree, Strongly disagree
How much do you agree or disagree with this statement: My skin’s complexion looks brighter, fresher after using the test product.	Strongly agree, Agree, Neither agree or disagree, Disagree, Strongly disagree
How much do you agree or disagree with this statement: My skin has a youthful, radiant glow after using the test product.	Strongly agree, Agree, Neither agree or disagree, Disagree, Strongly disagree
How much do you agree or disagree with this statement: I would like to continue using the test product.	Strongly agree, Agree, Neither agree or disagree, Disagree, Strongly disagree
How much do you agree or disagree with this statement: I would recommend the test product to my friends and family.	Strongly agree, Agree, Neither agree or disagree, Disagree, Strongly disagree
